# Exercise‐induced ventricular tachycardia in a case with hypertrophic cardiomyopathy taking cibenzoline

**DOI:** 10.1111/anec.12789

**Published:** 2020-08-19

**Authors:** Shinya Shiohira, Mihoko Kawabata, Masahiko Goya, Shingo Maeda, Tetsuo Sasano, Kenzo Hirao

**Affiliations:** ^1^ Department of Cardiovascular Medicine Tokyo Medical and Dental University Tokyo Japan; ^2^ Arrhythmia Advanced Therapy Center AOI Universal Hospital Kanagawa Japan

**Keywords:** hypertrophic cardiomyopathy, proarrhythmia, QRS duration, sodium channel blocker, use‐dependent, ventricular tachycardia

## Abstract

We report a 17‐year‐old woman with hypertrophic cardiomyopathy (HCM) successfully resuscitated from ventricular fibrillation while taking cibenzoline. During exercise–stress testing before implanting an implantable cardioverter–defibrillator, ventricular tachycardia was induced and thought to be a proarrhythmia due to the use‐dependent effect of the Na channel blockade with cibenzoline. In patients with arrhythmogenic substrates such as HCM, it is critical to pay attention to the proarrhythmic effects of class I antiarrhythmic drugs while increasing heart rate.

## 1 CASE REPORT

A 17‐year‐old woman with hypertrophic cardiomyopathy (HCM) was transferred to our hospital after successful resuscitation from ventricular fibrillation (VF). After the patient was diagnosed with HCM based on the echocardiogram and cardiac biopsy 2 years prior, she had been free from any symptoms or arrhythmic events while taking a beta‐blocking drug and ACE inhibitor. Both her father and grandfather had HCM, and her father died suddenly at 53 years old. Although she did not complain of anything, her BNP level was constantly high (500–970 pg/ml), so cibenzoline of 200 mg was added 3 months prior for the purpose of improvement of diastolic function caused by attenuation of the LV contraction and prevention of arrhythmic events. Then, she collapsed while walking due to VF.

She arrived at our hospital while undergoing therapeutic hypothermia. Her height was 159 cm and weight 56 kg. Her blood pressure was 158/89 mmHg. Physical examination revealed coarse crackles in her whole chest. Blood tests revealed normal renal function and electrolytes. The plasma cibenzoline concentration was not checked.

**FIGURE 1:**
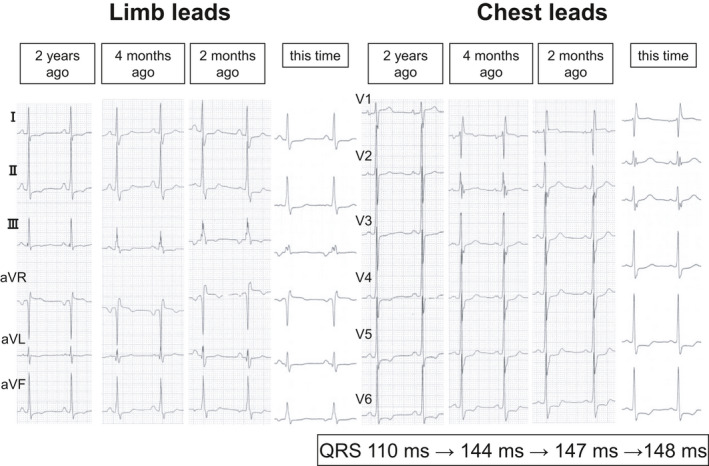
The serial ECGs from 2 years prior when she was diagnosed with hypertrophic cardiomyopathy, 4 months prior before the start of cibenzoline, 2 months prior with cibenzoline, and after her resuscitation. QRS duration gradually prolonged during those 2 years from 110 to 147 ms. However, it did not change between that before and after the start of cibenzoline

An ECG showed ectopic atrial rhythm with heart rate of 63 bpm, complete right bundle branch block, and left ventricular hypertrophy with ST‐T changes with QRS width of 148 ms and QTc of 0.514 (Figure 1). Those findings were almost like those before the episode. The serial ECGs showed that QRS width gradually prolonged over 2 years from 110 to 147 ms, suggesting that the conduction disturbance from the His–Purkinje system through the ventricular myocardium had been progressing (Figure 1). The ECG findings including QRS width did not change after commencement of the administration of cibenzoline.

**FIGURE 2:**
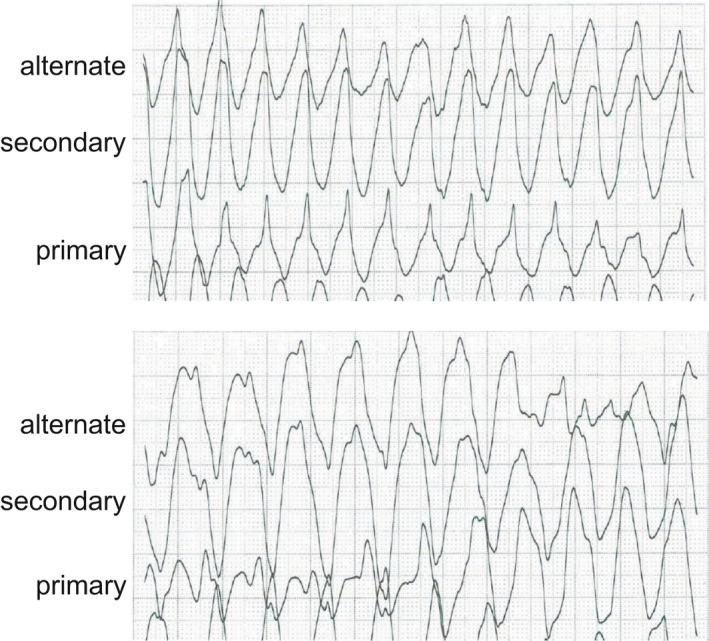
Ventricular tachycardia during screening for subcutaneous implantable cardioverter–defibrillators during treadmill exercise testing. Top panel: After 10’36’’ during the treadmill exercise testing, monomorphic ventricular tachycardia (VT) at 150 bpm accompanied the prolongation of the QRS duration. Bottom panel: After 1 min from the beginning of VT, QRS width during VT extended further, resulting in polymorphic VT causing the patient to collapse. In both panels, the ECG shows the alternate, secondary, and primary screening leads of the subcutaneous implantable cardioverter–defibrillator

**FIGURE 3:**
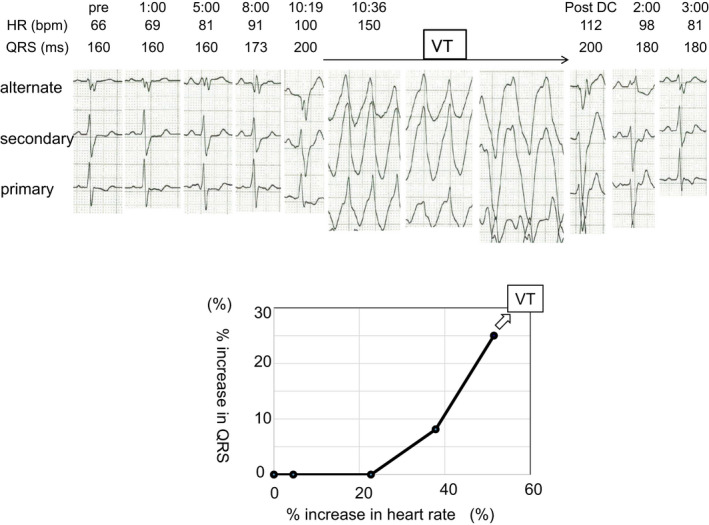
Top panel: The serial ECGs during treadmill exercise testing. The ECG shows the alternate, secondary, and primary screening leads of the subcutaneous implantable cardioverter–defibrillator. During exercise, the QRS duration increased progressively, accompanied by ventricular tachycardia (VT). After 3 DC shocks, VT slowed down and terminated. HR, heart rate; QRS, QRS duration; VT, ventricular tachycardia. Bottom panel: The rate of the increase in the QRS duration during treadmill exercise testing. All changes are expressed as a percent increase from the pre‐exercise baseline values. During exercise, the QRS duration increased progressively, accompanied by ventricular tachycardia

After she recovered almost fully without severe neurologic sequelae, a subcutaneous implantable cardioverter–defibrillator (S‐ICD) was selected for second prevention as she was young and pacing therapy was thought as unnecessary. The surface ECG screening prior to the S‐ICD implantation was performed. On the screening, the location of the surface electrodes was the fifth intercostal space along the left mid‐axillary line (left leg, LL), 1 cm lateral to the left sternal border and 1 cm above the xiphoid process (left arm, LA), and 14 cm cranial to electrode LA on the left parasternal line (right arm, RA). A ground electrode was placed on the right lower extremity. The alternate vector was from RA to LA electrode, primary vector from LA to LL electrode, and secondary vector from RA to LL electrode. All 3 vectors satisfied the S‐ICD screening template in both supine and upright positions; therefore, she was thought to be eligible for S‐ICD. Then, she underwent treadmill exercise testing for further screening for the S‐ICD with the 3 screening leads of the S‐ICD. During exercise, QRS duration increased progressively from 160 ms at rest at a heart rate of 66 bpm to 200 ms at peak exercise at a heart rate of 100 bpm, accompanied by monomorphic ventricular tachycardia (VT) at 150 bpm. QRS width during the VT extended further, resulting in polymorphic VT (Figures 2 and 3). After she had collapsed, the 3rd DC shock finally succeeded in terminating the VT. This VT was thought to be a proarrhythmia of cibenzoline, and therefore, cibenzoline was changed to sotalol. Re‐treadmill exercise testing while receiving sotalol revealed no change in QRS width during exercise, nor did it induce any VT at a similar exercise burden. Her S‐ICD was set with the primary vector as the sensing configuration, and she was well without any ICD shocks while taking sotalol.

## 2 DISCUSSION

Class I antiarrhythmic drugs are known to have frequency‐dependent effects on cardiac Na channels, causing the prolongation of the QRS complex accompanying an increase in the heart rate (Friedman & Stevenson, 1998; Ranger, Talajic, Lemery, Roy, & Nattel, 1989). The potent conduction‐slowing action might cause sine‐wave‐shaped VT particularly in patients with arrhythmogenic substrates. On the other hand, the proarrhythmic effect of class III antiarrhythmic drugs, K channel blockers, is known as torsade de pointes and is caused by QT prolongation (Friedman & Stevenson, 1998).

In this case with HCM who had been resuscitated from VF caused by cibenzoline, the prolongation of QRS duration during exercise with an increase in the heart rate resulted in polymorphic VT. In contrast, no increase in QRS duration or VT was observed during exercise while taking sotalol. Ranger *et al*. reported that exercise causes a rate‐dependent augmentation of the effects of flecainide, a class Ic antiarrhythmic drug, on the ventricular conduction by enhancing state‐dependent Na channel blockade and the patient with the greatest QRS increase developed monomorphic VT at peak exercise. They also reported that in patients receiving flecainide, the increase in QRS duration at rest was the most important determinant of the degree of further QRS prolongation resulting from exercise and that neither the flecainide dose nor serum flecainide concentration was predictors (Ranger et al., 1989). Class I agents have potent Na channel blockade effect, and their conduction‐slowing action might be related to proarrhythmias. When the patients have an underlying cardiac disease, such as HCM, the presence of arrhythmogenic substrate would tend to induce proarrhythmias easily. In our case, QRS duration progressively prolonged even before the initiation of cibenzoline indicating the progression of arrhythmogenic substrate; however, it did not prolong further at rest after receiving cibenzoline. It may mean that full evaluation of QRS duration including during exercise is required as QRS duration at rest might overlook the sign of proarrhythmia.

It is recommended as class IIa to combine disopyramide, a class Ia antiarrhythmic drug, with a beta‐blocking drug in the treatment of patients with obstructive HCM who do not respond to beta‐blocking drugs or verapamil alone (Gersh et al., 2011). Cibenzoline is also a class Ia antiarrhythmic drug and some reports have shown that it improves the LV diastolic dysfunction in the patients with both obstructive HCM and nonobstructive HCM (Hamada et al., 2001). However, the use of cibenzoline should be carefully considered in terms of its proarrhythmic effect. Not only routine 12‐lead ECG, but Holter monitoring or exercise testing to evaluate QRS duration during sinus tachycardia should be encouraged to forecast any proarrhythmias to save life from cibenzoline in the patients with HCM.

## 3 CONCLUSIONS

Special care using exercise testing and careful follow‐up are needed for the early detection of proarrhythmias due to cibenzoline, a sodium channel blocker, in patients with HCM despite of preserved systolic function.

## CONFLICTS OF INTEREST

None.

## AUTHOR'S CONTRIBUTION

All authors participate in writing and editing the manuscript.

## ETHICS

The authors have obtained the patient's informed consent.
